# Supplementary material for: Development of Large Hollow Particles for Pulmonary Delivery of Cyclosporine A
Source: Pharmaceutics. 2023 Aug 25;15(9):2204. doi: 10.3390/pharmaceutics15092204 (PMC10537410; doi:10.3390/pharmaceutics15092204)
Supplement: Supplementary file 1 [file pharmaceutics-15-02204-s001.zip › pharmaceutics-2565021-supplementary.pdf]

Supplementary Data

Development of Large Hollow Particles for Pulmonary Delivery of Cyclosporine A

(A)

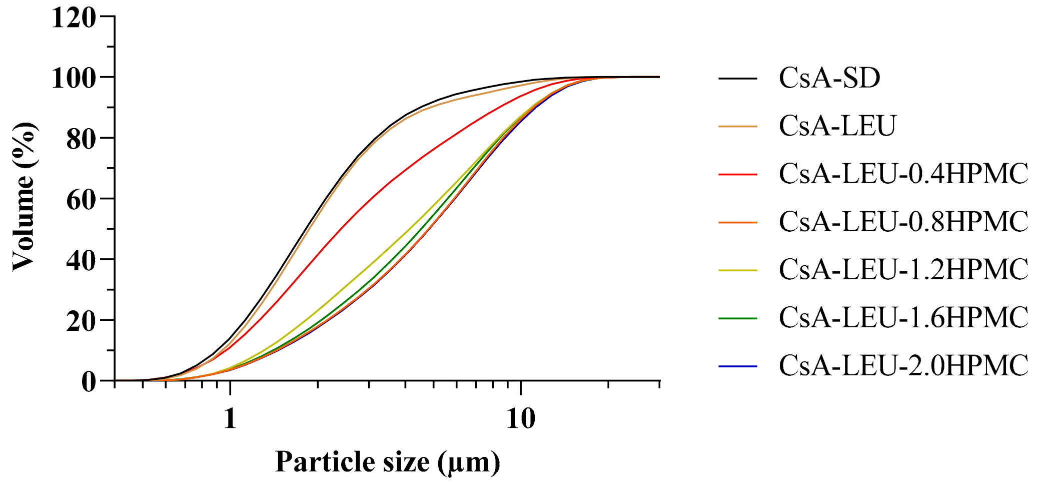

(B)

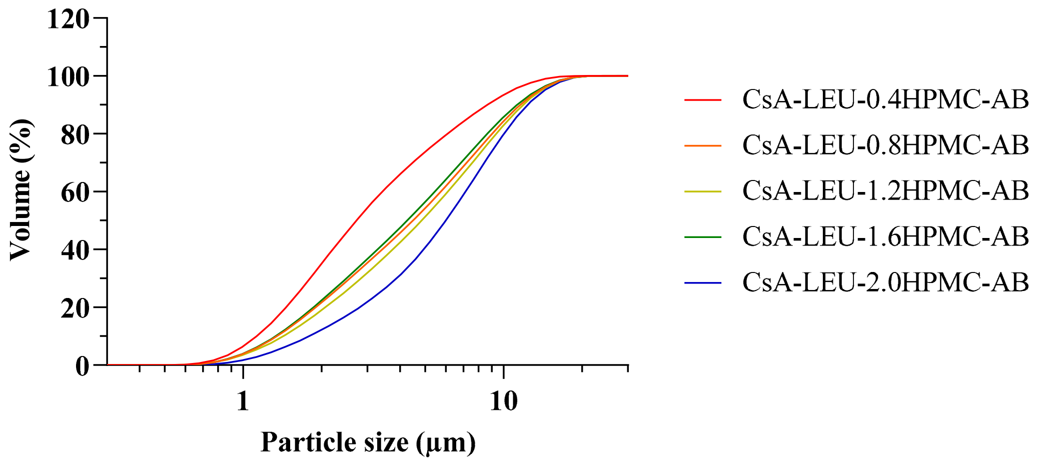

Figure S1. (A,B) Particle size distribution of the formulations.

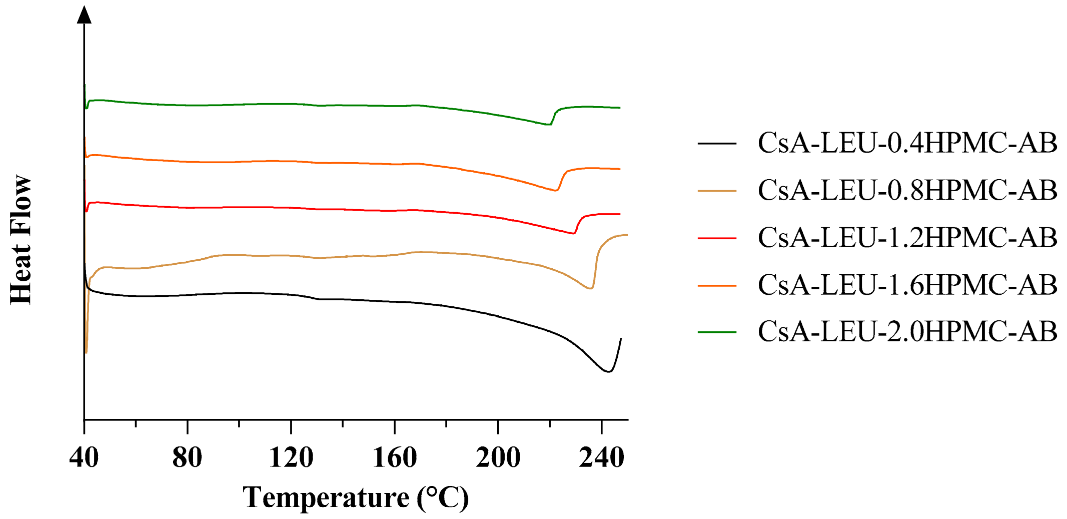

Figure S2. DSC curves of the formulations.
